# Oxidative Stress as a Therapeutic Target in Amyotrophic Lateral Sclerosis: Opportunities and Limitations

**DOI:** 10.3390/diagnostics11091546

**Published:** 2021-08-26

**Authors:** Hee Ra Park, Eun Jin Yang

**Affiliations:** KM Medicine Science Research Division, Korea Institute of Oriental Medicine (KIOM), Daejeon 34054, Korea; hrpark0109@kiom.re.kr

**Keywords:** amyotrophic lateral sclerosis, Lou Gehrig’s disease, motor neuron disease, oxidative stress, inflammation, reactive oxygen species, superoxide dismutase 1, antioxidants, therapeutics, phytochemicals

## Abstract

Amyotrophic lateral sclerosis (ALS), also known as motor neuron disease (MND) and Lou Gehrig’s disease, is characterized by a loss of the lower motor neurons in the spinal cord and the upper motor neurons in the cerebral cortex. Due to the complex and multifactorial nature of the various risk factors and mechanisms that are related to motor neuronal degeneration, the pathological mechanisms of ALS are not fully understood. Oxidative stress is one of the known causes of ALS pathogenesis. This has been observed in patients as well as in cellular and animal models, and is known to induce mitochondrial dysfunction and the loss of motor neurons. Numerous therapeutic agents have been developed to inhibit oxidative stress and neuroinflammation. In this review, we describe the role of oxidative stress in ALS pathogenesis, and discuss several anti-inflammatory and anti-oxidative agents as potential therapeutics for ALS. Although oxidative stress and antioxidant fields are meaningful approaches to delay disease progression and prolong the survival in ALS, it is necessary to investigate various animal models or humans with different subtypes of sporadic and familial ALS.

## 1. Introduction

Amyotrophic lateral sclerosis (ALS) is the most rapidly fatal motor neuron disease (MND), and is characterized by the degeneration of motor neurons in the cerebral cortex, brain stem, and spinal cord. Patients with ALS display progressive muscle atrophy, weakness, and loss of speech, leading to total or partial paralysis, and typically death 3–5 years after the onset of symptoms. According to the ALS Association, the average incidence of ALS worldwide is approximately 1/50,000 people per year. 

The cases of ALS are typically sporadic, but approximately 5–10% of cases are considered familial. Familial cases of ALS are linked by mutations in one of many different genes (*C9orf72*, *SOD1*, *TARDBP*, *FUS*, *VCP*, *ANG*, and *PFN1*). One of the most common genes that is mutated is superoxide dismutase 1 (*SOD1*), which encodes a Cu^2+^/Zn^2+^-binding SOD, and is directly associated with oxidative stress and inflammation. Although the causes of sporadic ALS remain unknown, various risk factors have been identified, including environmental factors (i.e., exposure to heavy metals, neurotoxicants, use of herbicides) [1], smoking, and age [2,3]. Nevertheless, sporadic ALS and familial ALS present similar clinical courses and neuropathologies [4]. Oxidative stress, accompanying motor neuronal death in ALS, causes disease pathogenesis, which is a common phenotype of sporadic ALS and familial ALS. A common phenotype of sporadic ALS and familial ALS is the extensive evidence of oxidative stress, which might be responsible for the degeneration or death of motor neurons that contribute to disease pathogenesis. Various molecular mechanisms of sporadic ALS and familial ALS have been suggested, including glutamate excitotoxicity, the dysregulation of neurotrophic factors, the disruption of axonal transport, protein misfolding and aggregation, inflammation, oxidative stress, and mitochondrial dysfunction [5,6,7,8,9,10,11,12]. 

## 2. Oxidative Stress and the Anti-Oxidative Defense System in ALS

Inflammation and oxidative stress are inter-dependent and closely associated in the pathogenesis of neurodegenerative diseases. Mild oxidative stress may protect the cells and tissues from infection and injury. However, severe oxidative stress induces chronic inflammation in the body, which, together, create a vicious cycle. Especially, in the brain, the activation of glial cells (astrocyte, microglia) in the central nervous system (CNS) and infiltration from peripheral immune cells are the major inducers of oxidative stress in the pathogenesis of the brain. Oxidative stress occurs early during disease onset and can exacerbate disease progression. Excessive oxidative stress-induced inflammation could have serious effects in neurodegenerative diseases, including Alzheimer’s disease, Parkinson’s disease, and ALS [13].

Oxidative stress is an imbalance of pro-oxidant (free radicals)/antioxidant homeostasis in the body, which can lead to cellular and tissue damage. These processes cause the production of free oxygen radicals or reactive oxygen species (ROS). These include hydrogen peroxide (H_2_O_2_), nitric oxide (NO), superoxide (O_2_^•−^), peroxide (O_2_^•−2^), hydroxyl ion (OH^−^), and reactive hydroxyl radicals (^•^OH), which are generated by multiple cellular processes [14]. ROS play important roles in both physiological and pathological conditions, because they can induce opposing effects on cellular metabolism and can act as beneficial or harmful molecules [15]. 

ROS are generated endogenously in the electron transport chain complex in the mitochondria, by oxidative phosphorylation, and during cellular defense processes against cytokine release and bacterial infections [16]. During oxidative phosphorylation, the leakage of electrons from the electron transport chains complex I and II leads to the reduction in oxygen to produce superoxide. Subsequently, superoxide radicals are quickly detoxicated to hydrogen peroxide, by the following two dismutases: the Cu^2+^/Zn^2+^-binding SOD (SOD1) in the cytosol and the mitochondrial intermembrane space, and manganese SOD (MnSOD or SOD2) in the mitochondrial matrix. In peroxisomes, catalase (CAT) and glutathione peroxidase (GSH-Px) reduce hydrogen peroxide to water and oxygen, while GSH-Px converts hydrogen peroxide to water and lipid peroxide [17]. Antioxidant enzymes, including CAT, GSH-Px, glutathione reductase (GR), and SOD, play important roles in the removal of ROS. Several oxidoreductases, including cyclooxygenase (COX), lipoxygenase (LOX), cytochrome P450 enzymes (CYP), and nicotinamide adenine dinucleotide phosphate oxidases (NOX), have been identified as major sources of cellular ROS production. NOX catalyzes the production of superoxide, by transferring electrons from NADPH to oxygen. 

## 3. Evidence for Oxidative Stress in ALS 

Oxidative stress has been shown to participate in a various diseases, including ALS, Alzheimer’s disease, Parkinson’s disease, and cancer, demonstrating the multiple mechanisms by which oxidants contribute to cellular damage and pathology. 

Many studies have reported increased oxidative stress in the pathogenesis of ALS. At the pathological level, the activation of astrocytes and microglia, as well as peripheral immune cells, induces neuroinflammation. These events contribute to ALS onset, progression, and pathogenesis. In the postmortem brains of patients with sporadic ALS and familial ALS, the markers of oxidative stress are increased [18]. Several studies have reported elevated levels of oxidative stress and inflammation markers in the serum, spinal cord, muscle, and brain of patients with ALS, as well as transgenic mouse models [19,20,21]. Increased 3-nitrotyrosine levels, a marker of oxidative damage mediated by peroxynitrite, were detected in both patients with sporadic and familial ALS, who also carry SOD1 mutations [22,23]. The levels of lipid peroxidation, including 4-hydroxynonenal (4-HNE) and 8-hydroxy-2′-deoxyguanosine (8-OHdG), also increased in the serum and cerebrospinal fluid of patients with ALS [24,25]. The levels of glutathione peroxidase (GPx) activity, SOD, GR, malondialdehyde (MDA), and 8-OHdG were significantly increased in the serum of patients with ALS, with significantly increased levels of 8-OHdG, MDA, and glutathione, and decreased total antioxidant levels [26]. Conversely, the levels of total antioxidant are increased in the serum of patients with ALS, suggesting that compensatory mechanisms protect against free radical toxicity and ALS pathogenesis [27]. Therefore, the regulation of cellular ROS levels is important for the prevention and treatment of ALS. Additionally, anti-oxidative activity could be an important therapeutic target for patients with ALS. 

In 1993, Rosen et al. first discovered mutations in the *SOD1* gene in the brains of patients with ALS [28]. Although it remains unclear why mutations in *SOD1* cause the degeneration of motor neurons, ALS transgenic mouse studies have shown that SOD1 mutations lead to a toxic gain of function. [29]. Furthermore, increased cellular oxidative stress and lipid peroxidation have been reported in an ALS, SOD1 mutant mouse model [30,31]. 

## 4. Oxidative Stress-Mediated Intracellular Signaling Pathways in ALS

At the molecular level, ROS can directly and/or indirectly modulate the mitogen-activated protein kinase (MAPK), nuclear factor erythroid-2-related factor-2 (Nrf2)/Keap1-antioxidant response element (ARE), and nuclear factor kappa B (NF-κB) intracellular signaling pathways, under pathological conditions, which contributes to disease progression. 

### 4.1. MAPK Signaling Pathway 

MAPK consists of the following four MAPKs: extracellular signal-related kinases (ERK1/2), c-Jun N-terminal kinases (JNK), p38 kinase (p38 MAPK), and big MAP kinase 1 (BMK1/ERK5). MAPK has been found to be activated in patients and animal models of neurodegenerative pathologies [32]. Inflammatory mediators induce the upregulation of MAPK, and activated MAPK promotes the release of more inflammatory mediators, which further increases neuroinflammation. Thus, this negative feedback loop causes uncontrolled neuroinflammation. 

Evidence suggests that the activation of MAPK, especially p38 MAPK, is closely associated with the degeneration of motor neurons and ALS pathogenesis. Dewil et al. found that p38 MAPK was significantly activated in the motor neurons and microglia of the ventral spinal cord of ALS SOD1^G93A^ transgenic mice, but these responses were completely inhibited by the p38 MAPK inhibitor, SB203580, and semapimod [33]. Furthermore, p38 MAPK and its upstream kinase, MAPK kinase 3–6 (MKK3–6), are also upregulated in the motor neurons of the lumbar spinal cord, and microglia are increased in transgenic mice carrying the SOD1^G93A^ mutation [34,35]. 

Slowed anterograde and retrograde axonal transport has been observed at the early stage of disease in both ALS mouse models and patients with ALS [36,37], and can cause motor neuron cell death. Axonal transport deficits are affected by the accumulation of abnormal phosphorylated neurofilaments in the axons of motor neurons, through the involvement of p38 MAPK. Direct evidence suggests that mutant SOD1 transgenic mice show increased p38 MAPK levels in the spinal cord, which directly activates kinesin-1, an anterograde axonal transport-related protein [38]. This is further supported by the ability of p38 MAPK inhibitors to restore retrograde axonal transport in primary motor neurons from SOD1^G93A^ mice [39]. 

Previous studies have demonstrated that p38 MAPK activation also occurs in iPSC-derived motor neurons and transgenic mice with mutations fused in sarcoma/translocated in liposarcoma (FUS) [40,41], which also causes familial ALS, due to toxic gain of function and the involvement of multifunctional proteins that are engaged in RNA and DNA processes [42,43]. Collectively, these reports indicate that p38 MAPK is linked to neuropathologies that are associated with ALS, and could be a promising therapeutic target for the treatment of ALS. 

### 4.2. The Nrf2/Keap1-ARE Signaling Pathway

The Nrf2/Keap1-ARE signaling pathway plays an important role in the regulation of cellular oxidative stress caused by ROS, and protects against cardiovascular diseases, cancer, and neurodegenerative diseases [44,45,46]. Kirby et al. reported that the expression of Nrf2 and its dependent genes dramatically decreased in the motor neurons of SOD1^G93A^ mice [47]. Another study also reported reductions in the Nrf2 levels in the motor neurons of the motor cortex, spinal cord, and lower limb muscles [48]. In contrast, the deletion of Nrf2 in SOD1^G93A^ mice accelerated motor neuron death and astrocyte activation, and caused earlier disease onset and shortened their life span [49]. These findings suggest that mutations in SOD1 lead to reductions in detoxifying enzymes and antioxidant response proteins, which are considered to be major cellular factors in ALS pathogenesis. 

### 4.3. NF-κB Signaling Pathway 

NF-κB regulates many important cellular responses, including inflammatory responses, cellular growth, and apoptosis. The NF-κB complex is composed of p50, p52, p65/RelA, c-Rel, and RelB. NF-κB activity is regulated by IkappaBs (IκBs), including IκB kinase-α (IKKα), IKKβ, and IKKγ. Various studies have reported that motor neuronal loss, and the activation of astrocytes and microglia, increase NF-κB signaling [50,51]. Neuron-specific suppression of IkB in SOD1^G93A^ mice and TDP-43 mice reduced motor neuron loss and reactive glia, by decreased misfolded SOD1 levels and TDP-43 translocation into the nucleus. These results ameliorate cognitive deficits and extend life span in ALS transgenic mice [52]. 

Several papers suggest that immune cells infiltrating the brain from the peripheral system play a pivotal role in neuromuscular disorders [53]. Activated peripheral macrophages are found in the motor neuron exon of ALS mouse models and patients with ALS [54]. In SOD1^G93A^ mice, the replacement of peripheral macrophages by cell-replacement technique reduced ROS and activation of peripheral macrophages and brain microglia [54]. The inhibition of NF-κB activation in the microglia and astrocytes reduces both brain and peripheral inflammation, and prolongs mouse survival [55,56]. 

## 5. Anti-Inflammatory and Anti-Oxidative Intervention for ALS 

The “one target, one drug” paradigm has proven to be difficult in providing effective therapeutic solutions for multifactorial diseases, such as ALS. Inflammation and oxidative stress are considered to be key pathogenic factors in ALS. Many studies have tested the therapeutic potential of various anti-inflammatory and anti-oxidative stress compounds for ALS. 

### 5.1. Synthetic Drug 

Riluzole (Rilutek), an anti-glutamatergic drug, is one of only two drugs that are approved for the treatment of ALS by the United States Food and Drug Administration, with its approval granted in 1995. Riluzole can delay disease symptoms and prolong survival for up to 3 months in patients [57]. Riluzole blocks glutamatergic neurotransmission in the CNS and inhibits glutamate release from the neurons in the brain. However, the anti-oxidative mechanism of riluzole has not been fully studied, because it has varied effects and complications on the inhibition of glutamate release. Nevertheless, several studies have reported that riluzole attenuates the oxidative injury that is induced by ALS pathogenesis in oxidative stress-induced neurons and SOD1^G93A^ neuronal cells [58,59]. Further, they also reported that riluzole has antioxidant properties in other disease models. Riluzole decreased cell death against tert-butyl hydroperoxide (t-BHP)-treated human retinal pigment epithelial cells [60]. Riluzole has neuroprotective effects on Alzheimer’s disease in rat models, due to decreased acetylcholinesterase (AChE) activity and oxidative stress marker [61]. In ALS patients, riluzole treatment reported increased survival and improved motor function [62,63]. 

Edaravone (Radicava) is a free radical scavenger that is used widely in the treatment of cerebral ischemia in Japan. It acts by reducing lipid peroxidation and hydroxyl radicals in the cerebral ischemic brain [64]. The anti-inflammatory and antioxidative effects of edaravone have been reported to reduce cytotoxicity, pro-inflammatory factors, and apoptotic cell death in neuroblastoma cells, under oxidative damage by hydrogen peroxide and amyloid beta 25–35 peptides, in a cellular model of Alzheimer’s disease, as well as hydrogen peroxide and lipopolysaccharide-induced rat astrocyte activation [65,66]. 

A recent study investigated the antioxidative efficacy of edaravone and its therapeutic effect on patients with ALS [67]. The anti-oxidative activity in serum and cerebrospinal fluid was reduced in patients with ALS, at an early disease stage (2 years of disease duration), which was reversed by edaravone treatment. Nagase et al. reported that increased plasma uric acid, 3-nitrotyrosine, and oxidized coenzyme Q10 were observed in 26 patients with ALS, whereas edaravone administration for 3 months (17 patients with ALS) and 6 months (13 patients with ALS) significantly decreased plasma free fatty acid and oxidative damage [68]. Furthermore, edaravone was also shown to modulate antioxidant-related intracellular signaling pathways, including MAPKs, Nrf2, and NF-κB, in various oxidative cellular and mouse models [69,70,71]. 

Masitinib is a novel tyrosine kinase inhibitor that targets microglia and mast cell-associated neuroinflammation, by inhibiting enzymes that are involved in inflammation [72]. Masitinib protects against motor nerve damage by inhibiting the proliferation and activation of microglia and mast cell-induced degranulation [73]. Masitinib was recently investigated in a phase 2/3 clinical trial (NCT02588677) that aimed to compare the efficacy and safety of masitinib in combination with riluzole, in patients with ALS [74]. This clinical trial reported that patients who were administered masitinib (3 mg/kg/day) and riluzole had improved ALS functional rating scale results, disease progression, and quality of life. 

EPI-589, a (R)-troloxamide quinone, is a safe and well-tolerated oxidoreductase enzyme inhibitor. The potential of EPI-589, as a therapy for ALS, has been shown in a phase 2a clinical trial (NCT02460679), wherein it significantly attenuated oxidative stress, neuroinflammation, and delayed disease progression. These trials suggest that the anti-oxidative and anti-inflammatory effects of synthetic drug candidates can be considered as interesting therapeutic strategies to modulate neuronal loss and neuroinflammation in patients with ALS, and thus require further investigation. 

### 5.2. Phytochemicals 

Phytochemicals are polyphenolic compounds that are present in plants that possess antioxidant and anti-inflammatory actions [75]. Several studies have reported that the administration or intake of phytochemicals reduces the risk of neurodegenerative diseases, through the modulation of oxidative stress [76,77]. 

The administration of 7,8-dihydroxyflavone (5 mg/kg, i.p.) reduced motor performance, and protected the total number of neurons and density of dendritic spines in the motor neurons of SOD1^G93A^ mice [78]. Fisetin (9 mg/kg, orally) delayed the development of motor deficits and reduced disease progression, resulting in an increased lifespan in SOD1^G93A^ mice [79]. Quercetin significantly decreased ROS in lymphoblast cell lines from patients with ALS, who carry SOD1 mutations [80]. Curcumin is the main constituent of *Curcuma longa*, a yellow pigment used as a spice and coloring agent. Its antioxidant properties have been evaluated in cell lines, mice, and patients [81]. Curcumin also has protective effects on motor neurons, against excitotoxicity and cytotoxicity [82,83]. The effects of resveratrol on ALS have been reported to decrease thimerosal-induced neurotoxicity in SH-SY5Y neuroblastoma cells and cortical neurons with SOD1^G93A^ overexpression, via the modulation of SIRT1/DREAM/PDYN, which are associated with neuronal death [84]. Resveratrol and MS-275, AMPK/sirtuin-1 activator, and HDAC inhibitors restored RelA acetylation and NF-κB-targeted genes, such as Bcl-xL and brain-derived neurotrophic factor (BDNF) in the spinal cord of SOD1^G93A^ mice, resulting in delayed disease onset and increased survival [85]. Additionally, the motor neurons of resveratrol-administered SOD1^G93A^ mice were protected by the activation of SIRT1/PGC-1a/p53 signaling and the restoration of mitochondrial biogenesis [85,86]. To determine the effects of curcumin on patients with ALS, a clinical trial is ongoing (NCT04654689), which is evaluating the effects of curcumin (200 mg) and resveratrol (75 mg) for at least 6 months. 

### 5.3. Cannabidiol and Cannabinoid (CB) Receptor 

Cannabidiol (CBD) is a phytocannabinoid in *Cannabis sativa* plants, and is known for its non-psychoactive, neuroprotective, anti-convulsive, and anti-oxidative activities [87]. CBD has been reported to have neuropharmacological effects on the brain in neurological disorders, including Alzheimer’s disease, Parkinson’s disease, multiple sclerosis, epilepsy, and ALS [88]. Several studies have suggested that CBD may act as a therapeutic agent for ALS. Nabiximol (Satives^®^) is an oral spray containing two chemicals, tetrahydrocannabinol (THC) and CBD that is used in the treatment of multiple sclerosis-related spasticity. According to human clinical trials, nabiximol has beneficial effects on motor neuron disease, by reducing spasticity symptoms [89]. Moreno-Martet et al. suggested that the a nabiximol-like combination of phytocannabinoids improved neurological scores, increased survival, and upregulated cannabinoid (CB) receptor 2 in SOD1^G93A^ mice [90]. CBD has also been shown to modulate ALS-linked genes in an in vitro system [91]. 

Furthermore, endocannabinoid systems, such as endocannabinoids and their selective receptors, play important roles in the CNS and protect against disease progression in ALS [92]. Endocannabinoids, including anandamide (AEA) and 2-arachidonoyl-glycerol (2-AG), are also targets of CB2 receptors, and modulate Nrf2 signaling, peroxisome proliferator-activated receptor-γ (PPAR-γ), and G-protein receptor 55 (GPR55) [93]. These processes are involved in attenuating glial reactivation, inflammation, and oxidative stress in neurodegenerative diseases. AEA and 2-AG levels were found to be increased in the lumbar section of the spinal cord of SOD1^G93A^ mice. As ALS-associated neurodegeneration begins in this section [94], this finding suggests that a defense mechanism exists in the spinal cord that involves the release of neurotoxins and cytokines by activated glia. Recently, Carter et al. reported a correlation between ALS and endocannabinoids in the serum of healthy adults and patients with ALS. AEA and the related lipids, 2-oleoylglycerol (2-OG), are positively associated with ALS progression, whereas 2-AG showed a negative association, suggesting that endocannabinoids and the related lipids can be used as biomarkers for the prediction of ALS, and are considered to be important endogenous cellular factors for the development of therapeutics for ALS. 

Experimental evidence, obtained from cellular systems and transgenic mouse models, has shown that the CB2 receptor and its agonist have therapeutic potential for ALS. CB2 receptor knockout mice showed accelerated motor neuronal death and premature mortality in TDP-43 mice [95]. Treatment with CB2 receptor agonists, such as AM1241, Win-55,212-2, and HU-308, reduced disease progression, motor dysfunction, paralysis, and reactive astrogliosis [96,97]. Additionally, the CB2 receptor showed neuroprotection in the reactive astrocytes of the spinal cord of dogs with degenerative myelopathy [98]. These findings support the potential of CBD-based drugs for disease treatment, and CB receptors as molecular targets for the treatment of ALS. 

### 5.4. Modification of Oxidative Stress-Related Molecules

NOX2 hyperactivation enhances inflammation and glial activation, which are related to disease progression in ALS [99]. NOX activity has been reported to be upregulated in the spinal cord and blood of mice and patients with ALS and SOD1 mutations [100,101]. Although the genetic deletion of NOX2 did not affect survival or neurochemical markers (GFAP, Iba-1, and CD68) in SOD1^G93A^ mice [101], pharmacological NOX inhibitors (perphenazine and thioridazine) could reduce NOX activity and superoxide levels in the spinal cord of SOD1^G93A^ mice [101]. The inactivation of NOX in double transgenic mice with SOD1^G93A^ and gp91^PHOX-^ increased survival and reduced pathological changes, such as microgliosis, and damaged myelinated axons in the spinal cord and motor neurons [102]. Apocynin, a NOX inhibitor, increased the number of motor neurons in the spinal cord and extended the lifespan of SOD1^G93A^ mice, through redox-sensitive Rac-1 regulation [103]. These reports suggest that specific NOX inhibitors may be potential preventive or therapeutic agents for ALS. 

Several research groups have demonstrated that the modulation of oxidative stress-related molecules, using gene therapy, alleviates disease symptoms and progression in ALS. Benkler et al. investigated the effect of a cocktail treatment, including EAAT2, GDH2, and Nrf2, using lentiviral vectors in SOD1^G93A^ mice, and showed an improvement in motor function and neurological function via anti-oxidative activity [104]. 

### 5.5. Vitamin E Supplementation 

The long-term intake of vitamins, especially vitamin E, could be associated with a lower risk of ALS [105]. Vitamin E is an important cellular antioxidant. Vitamin E intake has been shown to extend survival, and slow down disease onset and progression in SOD1^G93A^ mice [106]. In 2005 and 2007, case studies of patients with ALS reported that vitamin E supplementation was associated with a lower risk of death in patients with ALS, but did not affect the survival of patients with ALS [107,108]. Although the intake of vitamin E did not affect the survival in patients with ALS, oxidative stress markers in the plasma of patients with ALS were reduced after 3 months of combination treatment with riluzole and vitamin E [109]. Subsequently, combination treatments with vitamin E have been reported to have potential therapeutic properties, such as anti-oxidative ability and delayed disease progression, in patients with ALS [110]. 

## 6. The Limitations of Antioxidant Intervention for ALS

Oxidative stress is a component of various diseases, as well as ALS; therefore, the development of effective antioxidant interventions is very important for disease treatment. Nevertheless, the limitations of this concept require a clear consideration of whether oxidative stress is the primary (trigger for disease pathology) or secondary (contributor of disease progression) cause of ALS pathology, and an analysis of the pattern differences between sporadic ALS and familial ALS will also be needed.

Although various studies have reported that antioxidants and modified oxidative stress have beneficial effects on ALS, they fail in actual clinical trials, or have limited application on humans. Most cases of ALS are sporadic (about 90–95%), i.e., there is no genetic background or family history of the disease. About 5–10% of cases of ALS are familial, i.e., they have genetic causes and family histories. These classifications imply that ALS occurs mostly through a combination of genetic and non-genetic factors. Currently, there are only the following two drugs approved by the U.S. Food and Drug Administration for ALS treatment: riluzole and edaravone. However, they have some limitations. Riluzole extends survival for a few months (2–3 months), and edaravone is used in only a few countries and does not affect disease progression significantly. Even this is different for patients with familial ALS and sporadic ALS. Thus, various studies are being conducted to develop more effective therapeutic drugs for ALS. Nevertheless, it is difficult to develop a definitive drug, because the direct pathogenic mechanism of ALS has not been clearly elucidated, and various pathogenic factors and different cells are involved.

In the past decades, anti-ALS drug candidates have shown remarkable effects in pre-clinical studies and clinical trials; however, most clinical trials have failed or are ongoing. One of the reasons might be the limited use of animal models for evaluating drug tests. Although ALS is mostly sporadic, studies have employed familial types. The understanding of familial ALS is continually expanding, but that of other risk factors, such as environmental factors, lifestyle, or aging, remains poor. ALS transgenic animal models have been established using the well-known genetic mutations in ALS. It is not known how animal models represent the sporadic disease forms. Without a genetic target, animal models of sporadic diseases are limited. Furthermore, ALS is caused by various genetic mutations, besides SOD1 and TDP43, but animal models focus only on two genes. It is necessary to develop sporadic animal models and evaluate the efficacy of drug candidates accordingly.

Moreover, due to the differences between species, it is difficult to correlate the drug efficacy found in animal models to humans. Additionally, ALS pathogenesis involves multiple cell types contributing to disease onset and progression. However, so far, there is no model or mechanism covering all the causes. Recently, there has been active research on ALS pathogenesis, through induced pluripotent stem cells (iPSCs)-derived motor neurons from patients with ALS and personalized medicine.

## 7. Conclusions

Since ALS is a multifactorial and complex neurodegenerative disease, it will have clear limitations in drug development that simply targets a single etiological mechanism. In this review, we focused on the role of oxidative stress in the pathogenesis of ALS, as well as the cellular factors related to oxidative stress and antioxidant defense systems in ALS. Moreover, we summarized the therapeutic approaches with anti-oxidative activities in ALS treatments (Table 1). Based on previous research, oxidative stress is dramatically elevated in the serum, cerebrospinal fluid, motor neurons, spinal cord, and muscles of patients with ALS and animal models. These pieces of evidence suggest that oxidative stress could be a pathological mechanism involved in ALS pathogenesis and progression. However, this classification has many considerations, as the role of oxidative stress in ALS has not been fully elucidated. Nevertheless, a variety of approaches are needed to elucidate the pathogenesis of ALS and for the development of therapeutics that are suitable for multiple pathogenic mechanisms in ALS.

Overall, the present paper demonstrated that oxidative stress is closely related to the pathogenesis and disease mechanism in ALS. Although there are issues that need to be addressed, the modulation of oxidative stress is crucial in the development of ALS therapeutics. We expect that these considerations will contribute to the development of antioxidant therapeutics for ALS, and inform other perspectives to better understanding ALS.

## Figures and Tables

**Table 1:** Summary of the therapeutic interventions for anti-oxidant effects in ALS.

Category	Drug	Model	Treatment	Effects	References
Synthetic drug	Riluzole	SOD1^G93A^-expressed SH-SY5Y cells	1–10 μM, 24 h	(1) Protected the cell viability and reduced ROS/RNS levels (2) Direct antioxidant activity against oxidative stress	[59]
		959 ALS patients	50–242 mg/day	Prolonged survival of ALS patients at stage 4 (100 mg/day)	[62]
		974 ALS patients	50, 100, and 200 mg/day	(1) Increased survival (100 mg/day) (2) Small beneficial effect on bulbar and limb function	[63]
	Edaravone	22 ALS patients (disease duration of 2 years)	60 min/day, i.v. 2 weeks/2 weeks drug-free period/10 days	(1) Decreased anti-oxidative activity in serum and CSF (2) Improved ALS functional rating scale-revised (ALSFRS-R)	[67]
		26 ALS patients	17 patients (30 mg/day), 3 months and 13 continued treatments/6 months	(1) Increased plasma uric acid, 3-NT and oxidized CoQ10 (2) Decreased plasma free fatty acid and oxidative damages	[68]
		Nrf2 and SOD1^G93A^ double transgenic mice	15 mg/kg/day, 28 days, i.p.	(1) Accelerated oxidative stress in motor neuron of spinal cord and lower limb muscles (2) Enhanced oxidative stress in serum (3) Improved motor function	[70]
	Masitinib	SOD1^G93A^ rats	30 mg/kg/day, 15 days, p.o.	(1) Modulated microglia and mast cells activation (2) Prevented axonal pathology and the loss of myofibers	[73]
		Phase 2/3 clinical trial 394 ALS patients	Masitinib (3 or 4.5 mg/kg/day) with riluzole (100 mg/day)	(1) Improved ALS functional rating scale, disease progression and life quality (2) No change on efficacy and safety	NCT02588677 [74]
	EPI-589	Phase 2a clinical trial 19 ALS patients	500 mg/twice/day, 3 months	(1) Removed oxidative stress and neuroinflammation (2) Delayed disease progression	NCT02460679
Phytochemicals	7,8-DHF	SOD1^G93A^ mice	5 mg/kg, 3 days/week, 105 days	(1) Improved motor deficits (2) Prevented spinal motor neurons and dendritic spines	[78]
	Fisetin	SOD1^G93A^ mice	9 mg/kg/day, 10 weeks, p.o.	(1) Decreased free radical levels and human SOD1 levels (2) Increased antioxidant factors by ERK activation	[79]
	Quercetin	Lymphoblast cell lines derived from familial ALS patients	10 μM	Reduced ROS levels	[80]
	Curcumin	THP1 human monocytic cells with SOD1 mutant	1–500 μM, 6–72 h	(1) Inhibited the fibrillation of aggregated SOD1 by direct binding (2) Decreased cytotoxicity	[82]
		TDP-43^Q331K^ NSC-34 cells	15 μM, 24 h	Decreased abnormal excitability induced by TDP-43 mutation	[83]
	Resveratrol	SOD1^G93A^-expressed cortical neurons	0.01–3 μM, 24 h	Decreased neurotoxicity by SIRT1/DREAM/PDYN pathway	[84]
		SOD1^G93A^ mice	625 mg resveratrol-containing diet 30 days (25 mg/kg/day)	Increased life span, not motor performance by p53 deacetylation	[86]
Cannabidiol and endocannabinoid system	Nabiximols	Phase 2 clinical trial 60 ALS patients	Combination of 2.7 mg δ-THC and 2.5 mg CBD, 6 weeks, oromucosal spray	Reduced spasticity symptoms	[89]
	Phyto-cannabinoid	SOD1^G93A^ mice	20 mg/kg/day	(1) Improved neurological score (2) Increased mice survival and CB2 receptor	[90]
	Endocannabinoid	SOD1^G93A^ mice	35, 90 and 120 days of age	(1) Increased endocannabinoid levels in spinal cord with progressive degeneration (2) Decreased neuronal cell damage in spinal cord and neurotoxins by modulating glial activation	[94]
CB2 receptor	CB2 receptor knockout	TDP-43^A315T^ mice crossed with CB2 receptor knockout mice	7 weeks, 11 weeks, 65 days and 90 days of age	(1) Accelerated neurological deficits (motor function) (2) Induced severe motor neuron death and glial activation (3) Led to premature mortality	[95]
	Win-55,212-2 HU-308	SOD1^G93A^ mice and TDP-43^A315T^ mice	5 mg/kg/day, 30 days, i.p.	(1) Improved motor function (2) Protected neuronal loss and inhibited reactive gliosis	[96]
	AM1241	SOD1^G93A^ mice	1 mg/kg/day, i.p.	(1) Delayed motor impairment and disease progression (paralysis score and weight loss) (2) Increased mice survival	[96]
Modification of oxidative stress-related factors	NOX2 deletion	NOX2 knockout mice with SOD1^G93A^ mice	Perphenazine (3 mg/kg) Thioridazine (10 mg/kg)	(1) Increased NOX2 expression in microglia of SOD1^G93A^ mice and ALS patients (2) Decreased superoxide level and microglial activation	[101]
	NOX inactivation	SOD1^G93A^ mice crossed with gp91^PHOX^ knockout mice	At 1 month and 4-month-old mice	Delayed neurodegeneration and increased survival by modulation of IGF-1/Akt pathway	[102]
	NOX inhibitor (Apocynin)	SOD1^L8Q/G93A^-expressed SH-SY5Y cells and MO59J cells SOD1^G93A^ mice	100 μM 30, 150, 300 mg/kg/day Water intake	Attenuated the glial activation-induced cytotoxicity Increased life span	[103]
	Lentivirus cocktail (EAAT2, GDH2, Nrf2)	SOD1^G93A^ mice	65 days of age i.c., i.m.	(1) Reduced glutamate excitoxicity and improved anti-oxidant activity (2) Did not affect prolonged survival in mice (3) Prevented body weight loss, neurological score and motor function	[104]
Vitamin E	Vitamin E	SOD1^G93A^ mice	75 IU vitamin E-supplemented diet 5 months	Delayed disease progress, but does not prolong survival	[106]
	Clinical trial Human case study	289 ALS patients (less than 5 years duration)	500 mg/capsule and 50 mg riluzole 12 months	(1) After 3 months, decreased oxidative stress markers in plasma (2) Delayed disease progression (from milder state to severe state) (3) After 12 months, no effect on the survival	[109]

Abbreviations: 3-NT, 3-nitrotyrosine; 7,8-DHF, 7,8-dihydroxyflavone; ALS, amyotrophic lateral sclerosis; CB2, cannabinoid receptor 2; CoQ10, coenzyme Q10; CSF, cerebrospinal fluid; DREAM, downstream regulatory element antagonist modulator; EAAT2, excitatory amino acid transporter 2; GDH2, glutamate dehydrogenase 2; i.c., intracisternal; i.m., intramuscular; i.p., intraperitoneal; i.v., intravenous; IGF-1, insulin-like growth factor-1; NOX2, NADPH (nicotine adenine dinucleotide phosphate) oxidase 2; Nrf2, nuclear factor erythroid-2-related factor 2; NSC-34, neuroblastoma spinal cord-34; p.o., per oral; PDYN, prodynorphin; RNS, reactive nitrogen species; ROS, reactive oxygen species; SIRT1, sirtuin (silent mating-type information regulation 2 homolog) 1; SOD1, superoxide dismutase 1; TDP-43, TAR DNA-binding protein 43.

## Data Availability

The datasets supporting the conclusions in this study are contained in the article.

## References

[1] Bozzoni V., Pansarasa O., Diamanti L., Nosari G., Cereda C., Ceroni M. (2016). Amyotrophic lateral sclerosis and environmental factors. Funct. Neurol..

[2] Ferraro P.M., Cabona C., Meo G., Rolla-Bigliani C., Castellan L., Pardini M., Inglese M., Caponnetto C., Roccatagliata L. (2021). Age at symptom onset influences cortical thinning distribution and survival in amyotrophic lateral sclerosis. Neuroradiology.

[3] Zhan Y., Fang F. (2019). Smoking and amyotrophic lateral sclerosis: A mendelian randomization study. Ann. Neurol..

[4] Ajroud-Driss S., Siddique T. (2015). Sporadic and hereditary amyotrophic lateral sclerosis (ALS). Biochim. Biophys. Acta.

[5] Batra G., Jain M., Singh R.S., Sharma A.R., Singh A., Prakash A., Medhi B. (2019). Novel therapeutic targets for amyotrophic lateral sclerosis. Indian J. Pharmacol..

[6] Brambilla L., Martorana F., Guidotti G., Rossi D. (2018). Dysregulation of Astrocytic HMGB1 Signaling in Amyotrophic Lateral Sclerosis. Front. Neurosci..

[7] D’Ambrosi N., Cozzolino M., Carri M.T. (2018). Neuroinflammation in Amyotrophic Lateral Sclerosis: Role of Redox (dys)Regulation. Antioxid. Redox Signal..

[8] Dafinca R., Barbagallo P., Talbot K. (2021). The Role of Mitochondrial Dysfunction and ER Stress in TDP-43 and C9ORF72 ALS. Front. Cell. Neurosci..

[9] Granatiero V., Manfredi G. (2019). Mitochondrial Transport and Turnover in the Pathogenesis of Amyotrophic Lateral Sclerosis. Biology.

[10] Kefalakes E., Boselt S., Sarikidi A., Ettcheto M., Bursch F., Naujock M., Stanslowsky N., Schmuck M., Barenys M., Wegner F. (2019). Characterizing the multiple roles of FGF-2 in SOD1(G93A) ALS mice in vivo and in vitro. J. Cell. Physiol..

[11] Miranda-Lourenco C., Ribeiro-Rodrigues L., Fonseca-Gomes J., Tanqueiro S.R., Belo R.F., Ferreira C.B., Rei N., Ferreira-Manso M., de Almeida-Borlido C., Costa-Coelho T. (2020). Challenges of BDNF-based therapies: From common to rare diseases. Pharmacol. Res..

[12] Vucic S., Pavey N., Haidar M., Turner B.J., Kiernan M.C. (2021). Cortical hyperexcitability: Diagnostic and pathogenic biomarker of ALS. Neurosci. Lett..

[13] Piancone F., La Rosa F., Marventano I., Saresella M., Clerici M. (2021). The Role of the Inflammasome in Neurodegenerative. Dis. Mol..

[14] Coyle J.T., Puttfarcken P. (1993). Oxidative stress, glutamate, and neurodegenerative disorders. Science.

[15] Maldonado E., Rojas D.A., Morales S., Miralles V., Solari A. (2020). Dual and Opposite Roles of Reactive Oxygen Species (ROS) in Chagas Disease: Beneficial on the Pathogen and Harmful on the Host. Oxid. Med. Cell. Longev..

[16] Chapple I.L. (1997). Reactive oxygen species and antioxidants in inflammatory diseases. J. Clin. Periodontol..

[17] Halliwell B., Cross C.E. (1994). Oxygen-derived species: Their relation to human disease and environmental stress. Environ. Health Perspect..

[18] Agar J., Durham H. (2003). Relevance of oxidative injury in the pathogenesis of motor neuron diseases. Amyotroph. Lateral Scler. Other Mot. Neuron Disord..

[19] Ferrante R.J., Browne S.E., Shinobu L.A., Bowling A.C., Baik M.J., MacGarvey U., Kowall N.W., Brown R.H., Beal M.F. (1997). Evidence of increased oxidative damage in both sporadic and familial amyotrophic lateral sclerosis. J. Neurochem..

[20] Ryan T.E., Erickson M.L., Verma A., Chavez J., Rivner M.H., McCully K.K. (2014). Skeletal muscle oxidative capacity in amyotrophic lateral sclerosis. Muscle Nerve.

[21] Zhou J., Li A., Li X., Yi J. (2019). Dysregulated mitochondrial Ca(2+) and ROS signaling in skeletal muscle of ALS mouse model. Arch. Biochem. Biophys..

[22] Abe K., Pan L.H., Watanabe M., Kato T., Itoyama Y. (1995). Induction of nitrotyrosine-like immunoreactivity in the lower motor neuron of amyotrophic lateral sclerosis. Neurosci. Lett..

[23] Beal M.F., Ferrante R.J., Browne S.E., Matthews R.T., Kowall N.W., Brown R.H. (1997). Increased 3-nitrotyrosine in both sporadic and familial amyotrophic lateral sclerosis. Ann. Neurol..

[24] Simpson E.P., Henry Y.K., Henkel J.S., Smith R.G., Appel S.H. (2004). Increased lipid peroxidation in sera of ALS patients: A potential biomarker of disease burden. Neurology.

[25] Smith R.G., Henry Y.K., Mattson M.P., Appel S.H. (1998). Presence of 4-hydroxynonenal in cerebrospinal fluid of patients with sporadic amyotrophic lateral sclerosis. Ann. Neurol..

[26] Blasco H., Garcon G., Patin F., Veyrat-Durebex C., Boyer J., Devos D., Vourc’h P., Andres C.R., Corcia P. (2017). Panel of Oxidative Stress and Inflammatory Biomarkers in ALS: A Pilot Study. Can. J. Neurol Sci..

[27] Ilzecka J. (2003). Total antioxidant status is increased in the serum of amyotrophic lateral sclerosis patients. Scand. J. Clin. Lab. Investig..

[28] Rosen D.R., Siddique T., Patterson D., Figlewicz D.A., Sapp P., Hentati A., Donaldson D., Goto J., O’Regan J.P., Deng H.X. (1993). Mutations in Cu/Zn superoxide dismutase gene are associated with familial amyotrophic lateral sclerosis. Nature.

[29] Gurney M.E., Pu H., Chiu A.Y., Dal Canto M.C., Polchow C.Y., Alexander D.D., Caliendo J., Hentati A., Kwon Y.W., Deng H.X. (1994). Motor neuron degeneration in mice that express a human Cu, Zn superoxide dismutase mutation. Science.

[30] Tokuda E., Ono S., Ishige K., Naganuma A., Ito Y., Suzuki T. (2007). Metallothionein proteins expression, copper and zinc concentrations, and lipid peroxidation level in a rodent model for amyotrophic lateral sclerosis. Toxicology.

[31] Liu R., Li B., Flanagan S.W., Oberley L.W., Gozal D., Qiu M. (2002). Increased mitochondrial antioxidative activity or decreased oxygen free radical propagation prevent mutant SOD1-mediated motor neuron cell death and increase amyotrophic lateral sclerosis-like transgenic mouse survival. J. Neurochem..

[32] Ahmed T., Zulfiqar A., Arguelles S., Rasekhian M., Nabavi S.F., Silva A.S., Nabavi S.M. (2020). Map kinase signaling as therapeutic target for neurodegeneration. Pharmacol. Res..

[33] Dewil M., dela Cruz V.F., Van Den Bosch L., Robberecht W. (2007). Inhibition of p38 mitogen activated protein kinase activation and mutant SOD1(G93A)-induced motor neuron death. Neurobiol. Dis..

[34] Veglianese P., Lo Coco D., Bao Cutrona M., Magnoni R., Pennacchini D., Pozzi B., Gowing G., Julien J.P., Tortarolo M., Bendotti C. (2006). Activation of the p38MAPK cascade is associated with upregulation of TNF alpha receptors in the spinal motor neurons of mouse models of familial ALS. Mol. Cell. Neurosci..

[35] Tortarolo M., Veglianese P., Calvaresi N., Botturi A., Rossi C., Giorgini A., Migheli A., Bendotti C. (2003). Persistent activation of p38 mitogen-activated protein kinase in a mouse model of familial amyotrophic lateral sclerosis correlates with disease progression. Mol. Cell. Neurosci..

[36] Rouleau G.A., Clark A.W., Rooke K., Pramatarova A., Krizus A., Suchowersky O., Julien J.P., Figlewicz D. (1996). SOD1 mutation is associated with accumulation of neurofilaments in amyotrophic lateral sclerosis. Ann. Neurol..

[37] De Vos K.J., Hafezparast M. (2017). Neurobiology of axonal transport defects in motor neuron diseases: Opportunities for translational research?. Neurobiol. Dis..

[38] Morfini G.A., Bosco D.A., Brown H., Gatto R., Kaminska A., Song Y., Molla L., Baker L., Marangoni M.N., Berth S. (2013). Inhibition of fast axonal transport by pathogenic SOD1 involves activation of p38 MAP kinase. PLoS ONE.

[39] Gibbs K.L., Kalmar B., Rhymes E.R., Fellows A.D., Ahmed M., Whiting P., Davies C.H., Greensmith L., Schiavo G. (2018). Inhibiting p38 MAPK alpha rescues axonal retrograde transport defects in a mouse model of ALS. Cell Death Dis..

[40] Bhinge A., Namboori S.C., Zhang X., VanDongen A.M.J., Stanton L.W. (2017). Genetic Correction of SOD1 Mutant iPSCs 1Reveals ERK and JNK Activated AP1 as a Driver of Neurodegeneration in Amyotrophic Lateral Sclerosis. Stem Cell Rep..

[41] Sama R.R., Fallini C., Gatto R., McKeon J.E., Song Y., Rotunno M.S., Penaranda S., Abdurakhmanov I., Landers J.E., Morfini G. (2017). ALS-linked FUS exerts a gain of toxic function involving aberrant p38 MAPK activation. Sci. Rep..

[42] Vance C., Rogelj B., Hortobagyi T., De Vos K.J., Nishimura A.L., Sreedharan J., Hu X., Smith B., Ruddy D., Wright P. (2009). Mutations in FUS, an RNA processing protein, cause familial amyotrophic lateral sclerosis type 6. Science.

[43] Kwiatkowski T.J., Bosco D.A., Leclerc A.L., Tamrazian E., Vanderburg C.R., Russ C., Davis A., Gilchrist J., Kasarskis E.J., Munsat T. (2009). Mutations in the FUS/TLS gene on chromosome 16 cause familial amyotrophic lateral sclerosis. Science.

[44] Jaganjac M., Milkovic L., Sunjic S.B., Zarkovic N. (2020). The NRF2, Thioredoxin, and Glutathione System in Tumorigenesis and Anticancer Therapies. Antioxidants.

[45] Vashi R., Patel B.M. (2021). NRF2 in Cardiovascular Diseases: A Ray of Hope!. J. Cardiovasc. Transl. Res..

[46] Khan H., Tundis R., Ullah H., Aschner M., Belwal T., Mirzaei H., Akkol E.K. (2020). Flavonoids targeting NRF2 in neurodegenerative disorders. Food Chem. Toxicol..

[47] Kirby J., Halligan E., Baptista M.J., Allen S., Heath P.R., Holden H., Barber S.C., Loynes C.A., Wood-Allum C.A., Lunec J. (2005). Mutant SOD1 alters the motor neuronal transcriptome: Implications for familial ALS. Brain.

[48] Sarlette A., Krampfl K., Grothe C., Neuhoff N., Dengler R., Petri S. (2008). Nuclear erythroid 2-related factor 2-antioxidative response element signaling pathway in motor cortex and spinal cord in amyotrophic lateral sclerosis. J. Neuropathol. Exp. Neurol..

[49] Guo Y., Zhang Y., Wen D., Duan W., An T., Shi P., Wang J., Li Z., Chen X., Li C. (2013). The modest impact of transcription factor Nrf2 on the course of disease in an ALS animal model. Lab. Investig..

[50] Frakes A.E., Ferraiuolo L., Haidet-Phillips A.M., Schmelzer L., Braun L., Miranda C.J., Ladner K.J., Bevan A.K., Foust K.D., Godbout J.P. (2014). Microglia induce motor neuron death via the classical NF-kappaB pathway in amyotrophic lateral sclerosis. Neuron.

[51] Crosio C., Valle C., Casciati A., Iaccarino C., Carri M.T. (2011). Astroglial inhibition of NF-kappaB does not ameliorate disease onset and progression in a mouse model for amyotrophic lateral sclerosis (ALS). PLoS ONE.

[52] Dutta K., Thammisetty S.S., Boutej H., Bareil C., Julien J.P. (2020). Mitigation of ALS Pathology by Neuron-Specific Inhibition of Nuclear Factor Kappa B Signaling. J. Neurosci..

[53] Kallstig E., McCabe B.D., Schneider B.L. (2021). The Links between ALS and NF-kappaB. Int. J. Mol. Sci..

[54] Chiot A., Zaidi S., Iltis C., Ribon M., Berriat F., Schiaffino L., Jolly A., de la Grange P., Mallat M., Bohl D. (2020). Modifying macrophages at the periphery has the capacity to change microglial reactivity and to extend ALS survival. Nat. Neurosci..

[55] Ibarburu S., Kovacs M., Varela V., Rodriguez-Duarte J., Ingold M., Invernizzi P., Porcal W., Arevalo A.P., Perelmuter K., Bollati-Fogolin M. (2021). A Nitroalkene Benzoic Acid Derivative Targets Reactive Microglia and Prolongs Survival in an Inherited Model of ALS via NF-kappaB Inhibition. Neurotherapeutics.

[56] Dresselhaus E.C., Meffert M.K. (2019). Cellular Specificity of NF-kappaB Function in the Nervous System. Front. Immunol..

[57] Hugon J. (1996). Riluzole and ALS therapy. Wien. Med. Wochenschr..

[58] Noh K.M., Hwang J.Y., Shin H.C., Koh J.Y. (2000). A novel neuroprotective mechanism of riluzole: Direct inhibition of protein kinase C. Neurobiol. Dis..

[59] Sala G., Arosio A., Conti E., Beretta S., Lunetta C., Riva N., Ferrarese C., Tremolizzo L. (2019). Riluzole Selective Antioxidant Effects in Cell Models Expressing Amyotrophic Lateral Sclerosis Endophenotypes. Clin. Psychopharmacol. Neurosci..

[60] Shen C., Ma W., Zheng W., Huang H., Xia R., Li C., Zhu X. (2017). The antioxidant effects of riluzole on the APRE-19 celll model injury-induced by t-BHP. BMC Ophthalmol..

[61] Mokhtari Z., Baluchnejadmojarad T., Nikbakht F., Mansouri M., Roghani M. (2017). Riluzole ameliorates learning and memory deficits in Abeta25-35-induced rat model of Alzheimer’s disease and is independent of cholinoceptor activation. Biomed. Pharmacother..

[62] Fang T., Khleifat A.A., Meurgey J., Jones A., Leigh P.N., Bensimon G., Al-Chalabi A. (2018). Stage at which riluzole treatment prolongs survival in patients with amyotrophic lateral sclerosis: A retrospective analysis of data from a dose-ranging study. Lancet Neurol..

[63] Miller R.G., Mitchell J.D., Moore D.H. (2012). Riluzole for amyotrophic lateral sclerosis (ALS)/motor neuron disease (MND). Cochrane Database Syst. Rev..

[64] Yamamoto T., Yuki S., Watanabe T., Mitsuka M., Saito K.I., Kogure K. (1997). Delayed neuronal death prevented by inhibition of increased hydroxyl radical formation in a transient cerebral ischemia. Brain Res..

[65] Guo Z., Wu H.T., Li X.X., Yu Y., Gu R.Z., Lan R., Qin X.Y. (2020). Edaravone protects rat astrocytes from oxidative or neurotoxic inflammatory insults by restoring Akt/Bcl-2/Caspase-3 signaling axis. IBRO Rep..

[66] Jami M.S., Salehi-Najafabadi Z., Ahmadinejad F., Hoedt E., Chaleshtori M.H., Ghatrehsamani M., Neubert T.A., Larsen J.P., Moller S.G. (2015). Edaravone leads to proteome changes indicative of neuronal cell protection in response to oxidative stress. Neurochem. Int..

[67] Ohta Y., Yamashita T., Nomura E., Hishikawa N., Ikegami K., Osakada Y., Matsumoto N., Kawahara Y., Yunoki T., Takahashi Y. (2020). Improvement of a decreased anti-oxidative activity by edaravone in amyotrophic lateral sclerosis patients. J. Neurol. Sci..

[68] Nagase M., Yamamoto Y., Miyazaki Y., Yoshino H. (2016). Increased oxidative stress in patients with amyotrophic lateral sclerosis and the effect of edaravone administration. Redox Rep..

[69] Jangra A., Kwatra M., Singh T., Pant R., Kushwah P., Ahmed S., Dwivedi D., Saroha B., Lahkar M. (2016). Edaravone alleviates cisplatin-induced neurobehavioral deficits via modulation of oxidative stress and inflammatory mediators in the rat hippocampus. Eur. J. Pharmacol..

[70] Ohta Y., Nomura E., Shang J., Feng T., Huang Y., Liu X., Shi X., Nakano Y., Hishikawa N., Sato K. (2019). Enhanced oxidative stress and the treatment by edaravone in mice model of amyotrophic lateral sclerosis. J. Neurosci. Res..

[71] Zhang L., Guo Y., Wang H., Zhao L., Ma Z., Li T., Liu J., Sun M., Jian Y., Yao L. (2019). Edaravone reduces Abeta-induced oxidative damage in SH-SY5Y cells by activating the Nrf2/ARE signaling pathway. Life Sci..

[72] Palomo V., Nozal V., Rojas-Prats E., Gil C., Martinez A. (2021). Protein kinase inhibitors for amyotrophic lateral sclerosis therapy. Br. J. Pharmacol..

[73] Trias E., King P.H., Si Y., Kwon Y., Varela V., Ibarburu S., Kovacs M., Moura I.C., Beckman J.S., Hermine O. (2018). Mast cells and neutrophils mediate peripheral motor pathway degeneration in ALS. JCI Insight.

[74] Mora J.S., Genge A., Chio A., Estol C.J., Chaverri D., Hernandez M., Marin S., Mascias J., Rodriguez G.E., Povedano M. (2020). Masitinib as an add-on therapy to riluzole in patients with amyotrophic lateral sclerosis: A randomized clinical trial. Amyotroph. Lateral Scler. Front. Degener..

[75] Rasool M., Malik A., Qureshi M.S., Manan A., Pushparaj P.N., Asif M., Qazi M.H., Qazi A.M., Kamal M.A., Gan S.H. (2014). Recent updates in the treatment of neurodegenerative disorders using natural compounds. Evid. Based Complement. Altern. Med..

[76] Truzzi F., Tibaldi C., Zhang Y., Dinelli G., D′Amen E. (2021). An Overview on Dietary Polyphenols and Their Biopharmaceutical Classification System (BCS). Int. J. Mol. Sci..

[77] Fujita K., Yamafuji M., Nakabeppu Y., Noda M. (2012). Therapeutic approach to neurodegenerative diseases by medical gases: Focusing on redox signaling and related antioxidant enzymes. Oxid. Med. Cell. Longev..

[78] Korkmaz O.T., Aytan N., Carreras I., Choi J.K., Kowall N.W., Jenkins B.G., Dedeoglu A. (2014). 7,8-Dihydroxyflavone improves motor performance and enhances lower motor neuronal survival in a mouse model of amyotrophic lateral sclerosis. Neurosci. Lett..

[79] Wang T.H., Wang S.Y., Wang X.D., Jiang H.Q., Yang Y.Q., Wang Y., Cheng J.L., Zhang C.T., Liang W.W., Feng H.L. (2018). Fisetin Exerts Antioxidant and Neuroprotective Effects in Multiple Mutant hSOD1 Models of Amyotrophic Lateral Sclerosis by Activating ERK. Neuroscience.

[80] Said Ahmed M., Hung W.Y., Zu J.S., Hockberger P., Siddique T. (2000). Increased reactive oxygen species in familial amyotrophic lateral sclerosis with mutations in SOD1. J. Neurol. Sci..

[81] Zia A., Farkhondeh T., Pourbagher-Shahri A.M., Samarghandian S. (2021). The role of curcumin in aging and senescence: Molecular mechanisms. Biomed. Pharmacother..

[82] Bhatia N.K., Srivastava A., Katyal N., Jain N., Khan M.A., Kundu B., Deep S. (2015). Curcumin binds to the pre-fibrillar aggregates of Cu/Zn superoxide dismutase (SOD1) and alters its amyloidogenic pathway resulting in reduced cytotoxicity. Biochim. Biophys. Acta.

[83] Dong H., Xu L., Wu L., Wang X., Duan W., Li H., Li C. (2014). Curcumin abolishes mutant TDP-43 induced excitability in a motoneuron-like cellular model of ALS. Neuroscience.

[84] Laudati G., Mascolo L., Guida N., Sirabella R., Pizzorusso V., Bruzzaniti S., Serani A., Di Renzo G., Canzoniero L.M.T., Formisano L. (2019). Resveratrol treatment reduces the vulnerability of SH-SY5Y cells and cortical neurons overexpressing SOD1-G93A to Thimerosal toxicity through SIRT1/DREAM/PDYN pathway. Neurotoxicology.

[85] Schiaffino L., Bonafede R., Scambi I., Parrella E., Pizzi M., Mariotti R. (2018). Acetylation state of RelA modulated by epigenetic drugs prolongs survival and induces a neuroprotective effect on ALS murine model. Sci. Rep..

[86] Markert C.D., Kim E., Gifondorwa D.J., Childers M.K., Milligan C.E. (2010). A single-dose resveratrol treatment in a mouse model of amyotrophic lateral sclerosis. J. Med. Food.

[87] Navarrete F., Garcia-Gutierrez M.S., Gasparyan A., Austrich-Olivares A., Manzanares J. (2021). Role of Cannabidiol in the Therapeutic Intervention for Substance Use Disorders. Front. Pharmacol..

[88] Melas P.A., Scherma M., Fratta W., Cifani C., Fadda P. (2021). Cannabidiol as a Potential Treatment for Anxiety and Mood Disorders: Molecular Targets and Epigenetic Insights from Preclinical Research. Int. J. Mol. Sci..

[89] Riva N., Mora G., Soraru G., Lunetta C., Ferraro O.E., Falzone Y., Leocani L., Fazio R., Comola M., Comi G. (2019). Safety and efficacy of nabiximols on spasticity symptoms in patients with motor neuron disease (CANALS): A multicentre, double-blind, randomised, placebo-controlled, phase 2 trial. Lancet Neurol..

[90] Moreno-Martet M., Espejo-Porras F., Fernandez-Ruiz J., de Lago E. (2014). Changes in endocannabinoid receptors and enzymes in the spinal cord of SOD1(G93A) transgenic mice and evaluation of a Sativex((R))-like combination of phytocannabinoids: Interest for future therapies in amyotrophic lateral sclerosis. CNS Neurosci. Ther..

[91] Rajan T.S., Scionti D., Diomede F., Grassi G., Pollastro F., Piattelli A., Cocco L., Bramanti P., Mazzon E., Trubiani O. (2017). Gingival Stromal Cells as an In Vitro Model: Cannabidiol Modulates Genes Linked with Amyotrophic Lateral Sclerosis. J. Cell Biochem..

[92] Rodriguez-Cueto C., Garcia-Toscano L., Santos-Garcia I., Gomez-Almeria M., Gonzalo-Consuegra C., Espejo-Porras F., Fernandez-Ruiz J., de Lago E. (2021). Targeting the CB2 receptor and other endocannabinoid elements to delay disease progression in amyotrophic lateral sclerosis. Br. J. Pharmacol..

[93] Paloczi J., Varga Z.V., Hasko G., Pacher P. (2018). Neuroprotection in Oxidative Stress-Related Neurodegenerative Diseases: Role of Endocannabinoid System Modulation. Antioxid. Redox Signal..

[94] Witting A., Weydt P., Hong S., Kliot M., Moller T., Stella N. (2004). Endocannabinoids accumulate in spinal cord of SOD1 G93A transgenic mice. J. Neurochem..

[95] Rodriguez-Cueto C., Gomez-Almeria M., Garcia Toscano L., Romero J., Hillard C.J., de Lago E., Fernandez-Ruiz J. (2021). Inactivation of the CB2 receptor accelerated the neuropathological deterioration in TDP-43 transgenic mice, a model of amyotrophic lateral sclerosis. Brain Pathol..

[96] Espejo-Porras F., Garcia-Toscano L., Rodriguez-Cueto C., Santos-Garcia I., de Lago E., Fernandez-Ruiz J. (2019). Targeting glial cannabinoid CB2 receptors to delay the progression of the pathological phenotype in TDP-43 (A315T) transgenic mice, a model of amyotrophic lateral sclerosis. Br. J. Pharmacol..

[97] Kim K., Moore D.H., Makriyannis A., Abood M.E. (2006). AM1241, a cannabinoid CB2 receptor selective compound, delays disease progression in a mouse model of amyotrophic lateral sclerosis. Eur. J. Pharmacol..

[98] Fernandez-Trapero M., Espejo-Porras F., Rodriguez-Cueto C., Coates J.R., Perez-Diaz C., de Lago E., Fernandez-Ruiz J. (2017). Upregulation of CB2 receptors in reactive astrocytes in canine degenerative myelopathy, a disease model of amyotrophic lateral sclerosis. Dis. Model. Mech..

[99] Pasinelli P., Brown R.H. (2006). Molecular biology of amyotrophic lateral sclerosis: Insights from genetics. Nat. Rev. Neurosci..

[100] Marrali G., Casale F., Salamone P., Fuda G., Caorsi C., Amoroso A., Brunetti M., Restagno G., Barberis M., Bertuzzo D. (2014). NADPH oxidase (NOX2) activity is a modifier of survival in ALS. J. Neurol..

[101] Seredenina T., Nayernia Z., Sorce S., Maghzal G.J., Filippova A., Ling S.C., Basset O., Plastre O., Daali Y., Rushing E.J. (2016). Evaluation of NADPH oxidases as drug targets in a mouse model of familial amyotrophic lateral sclerosis. Free Radic. Biol. Med..

[102] Wu D.C., Re D.B., Nagai M., Ischiropoulos H., Przedborski S. (2006). The inflammatory NADPH oxidase enzyme modulates motor neuron degeneration in amyotrophic lateral sclerosis mice. Proc. Natl. Acad. Sci. USA.

[103] Harraz M.M., Marden J.J., Zhou W., Zhang Y., Williams A., Sharov V.S., Nelson K., Luo M., Paulson H., Schoneich C. (2008). SOD1 mutations disrupt redox-sensitive Rac regulation of NADPH oxidase in a familial ALS model. J. Clin. Investig..

[104] Benkler C., Barhum Y., Ben-Zur T., Offen D. (2016). Multifactorial Gene Therapy Enhancing the Glutamate Uptake System and Reducing Oxidative Stress Delays Symptom Onset and Prolongs Survival in the SOD1-G93A ALS Mouse Model. J. Mol. Neurosci..

[105] Wang H., O’Reilly E.J., Weisskopf M.G., Logroscino G., McCullough M.L., Schatzkin A., Kolonel L.N., Ascherio A. (2011). Vitamin E intake and risk of amyotrophic lateral sclerosis: A pooled analysis of data from 5 prospective cohort studies. Am. J. Epidemiol..

[106] Gurney M.E., Cutting F.B., Zhai P., Doble A., Taylor C.P., Andrus P.K., Hall E.D. (1996). Benefit of vitamin E, riluzole, and gabapentin in a transgenic model of familial amyotrophic lateral sclerosis. Ann. Neurol..

[107] Ricciarelli R., Argellati F., Pronzato M.A., Domenicotti C. (2007). Vitamin E and neurodegenerative diseases. Mol. Asp. Med..

[108] Ascherio A., Weisskopf M.G., O’Reilly E.J., Jacobs E.J., McCullough M.L., Calle E.E., Cudkowicz M., Thun M.J. (2005). Vitamin E intake and risk of amyotrophic lateral sclerosis. Ann. Neurol..

[109] Desnuelle C., Dib M., Garrel C., Favier A. (2001). A double-blind, placebo-controlled randomized clinical trial of alpha-tocopherol (vitamin E) in the treatment of amyotrophic lateral sclerosis. ALS riluzole-tocopherol Study Group. Amyotroph. Lateral Scler. Other Mot. Neuron Disord..

[110] Michal Freedman D., Kuncl R.W., Weinstein S.J., Malila N., Virtamo J., Albanes D. (2013). Vitamin E serum levels and controlled supplementation and risk of amyotrophic lateral sclerosis. Amyotroph. Lateral Scler. Front. Degener..

